# MT2A is an early predictive biomarker of response to chemotherapy and a potential therapeutic target in osteosarcoma

**DOI:** 10.1038/s41598-019-48846-2

**Published:** 2019-08-23

**Authors:** Adèle Mangelinck, Maria Eugénia Marques da Costa, Bojana Stefanovska, Olivia Bawa, Mélanie Polrot, Nathalie Gaspar, Olivia Fromigué

**Affiliations:** 1 INSERM, UMR981, Gustave Roussy, Villejuif, F-94805 France; 2 Ecole Nationale Supérieure de Chimie de Montpellier (ENSCM), Montpellier, F-34090 France; 3 Université de Montpellier, Montpellier, F-34090 France; 4 CNRS, UMR8203, Gustave Roussy, Villejuif, F-94805 France; 5 Université Paris Sud, Université Paris Saclay, Orsay, F-91400 France; 6 CESAM, Department of Biology, University of Aveiro, Aveiro, P-3810 Portugal; 7 Plateforme d’évaluation préclinique (PFEP), Gustave Roussy, Villejuif, F-94805 France; 8 Département de cancérologie de l’enfant et de l’adolescent, Gustave Roussy, Villejuif, F-94805 France

**Keywords:** Tumour biomarkers, Bone cancer

## Abstract

Osteosarcoma is the most prevalent primary bone malignancy in children and young adults. Resistance to chemotherapy remains a key challenge for effective treatment of patients with osteosarcoma. The aim of the present study was to investigate the preventive role of metallothionein-2A (MT2A) in response to cytotoxic effects of chemotherapy. A panel of human and murine osteosarcoma cell lines, modified for MT2A were evaluated for cell viability, and motility (wound healing assay). Cell-derived xenograft models were established in mice. FFPE tumour samples were assessed by IHC. *In vitro* experiments indicated a positive correlation between half-maximal inhibitory concentration (IC50) for drugs in clinical practice, and MT2A mRNA level. This reinforced our previously reported correlation between MT2A mRNA level in tumour samples at diagnosis and overall survival in patients with osteosarcoma. In addition, MT2A/MT2 silencing using shRNA strategy led to a marked reduction of IC50 values and to enhanced cytotoxic effect of chemotherapy on primary tumour. Our results show that MT2A level could be used as a predictive biomarker of resistance to chemotherapy, and provide with preclinical rational for MT2A targeting as a therapeutic strategy for enhancing anti-tumour treatment of innate chemo-resistant osteosarcoma cells.

## Introduction

Osteosarcoma is the most common primary malignant bone tumour, with high incidence in children, adolescents and young adults^1^. The current protocols combine surgery to neoadjuvant and adjuvant chemotherapy regimen^2^, leading to a five-year overall survival rate levelled off around 60–70% over the past fifty years^3^. The major obstacles for a more favourable outcome are the frequent occurrence of drug resistance, and metastatic relapses. The underlying molecular mechanisms for chemo-resistance remain multiple and unclear, as well as their possible connection with the metastatic phenotype.

Metallothioneins (MTs) are a group of cysteine-rich metal-binding proteins exhibiting significant chelating properties^4,5^. Hence, MTs play a key role in trace elements (zinc, copper…) homeostasis, protection against oxidative stress, and toxic heavy metals. Such detoxication process can decrease cell vulnerability to anticancer drugs, and modulate cancer cells response to chemotherapy (reviewed in^6^). We previously reported that high MT2A mRNA level in tumour tissue at diagnosis correlates with poor response to chemotherapy and poorer outcome in a small cohort of osteosarcoma patients, suggesting a prognostic significance^7^.

The present study first assesses the predictive value of MT2A expression level in response to various chemotherapy agents using a panel of osteosarcoma cell lines. Then, the potential value of MT2A repression to reinforce chemotherapy cytotoxicity is assessed using *in vitro* and preclinical models.

## Results

### MT2A mRNA level correlates with chemotherapy IC50 values

**Figure 1:**
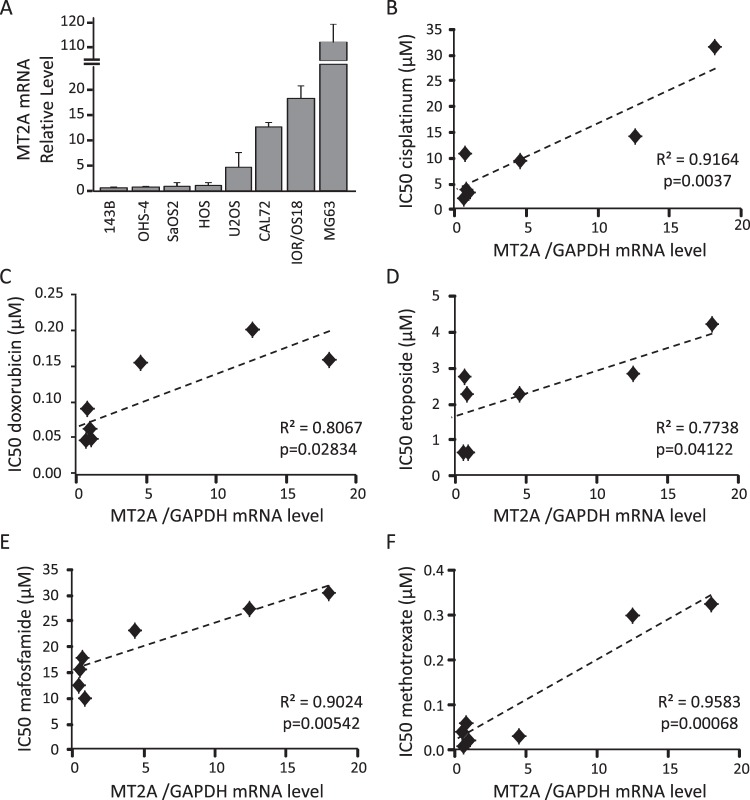
Correlation between MT2A mRNA level and IC50 values for chemotherapeutic drugs. (**A**) Expression pattern of MT2A in a panel of osteosarcoma cell lines, as assessed by RT-qPCR. GAPDH was used as internal reference gene. The relative mRNA level was calculated using the 2^−ΔΔCT^ method and expressed as mean ± standard deviation (n = 3–5). (**B**–**F**) Spearman correlation between the MT2A mRNA level and half maximal inhibitory concentration (IC50) values determined for cisplatinum (**B**), doxorubicin (**C**), etoposide (**D**), mafosfamide (**E**) and methotrexate (**F**). The black line shows the regression line. The determination coefficient R² and the significance of the F test (P-value) are also given.

We first determined the expression level of MT2A in a panel of eight osteosarcoma cell lines by real-time quantitative RT-PCR. Cell lines exhibited various basal expression level of MT2A mRNA (Fig. 1A). We then determined the dose-response to five currently used chemotherapeutic drugs, namely cisplatinum, doxorubicin, etoposide, mafosfamide and methotrexate. Cells in exponential phase of growth were exposed to the indicated drugs for 72 hrs, and cell viability was assessed by the MTS test. All tested drugs dose-dependently reduced cell viability, but cell lines exhibited various sensitivity to a given drug, as reflected by half maximal inhibitory concentration (IC50) values (Supplemental Table 2). IC50 values are consistent with those referenced in^8^.

We then determined the correlation between MT2A mRNA level and IC50 values for each chemotherapeutic drug (Fig. 1B–F). MG63 cell line was removed from the correlation analysis because of its huge basal MT2A expression. A strong positive correlation was detected between MT2A level and resistance to all tested drugs (R > 0.7, p < 0.05).

Taken together, these results indicate that MT2A mRNA level correlate with cell resistance to chemotherapy-based therapies currently used in osteosarcoma.

### Chemotherapy treatment induces MT2A mRNA expression

**Figure 2:**
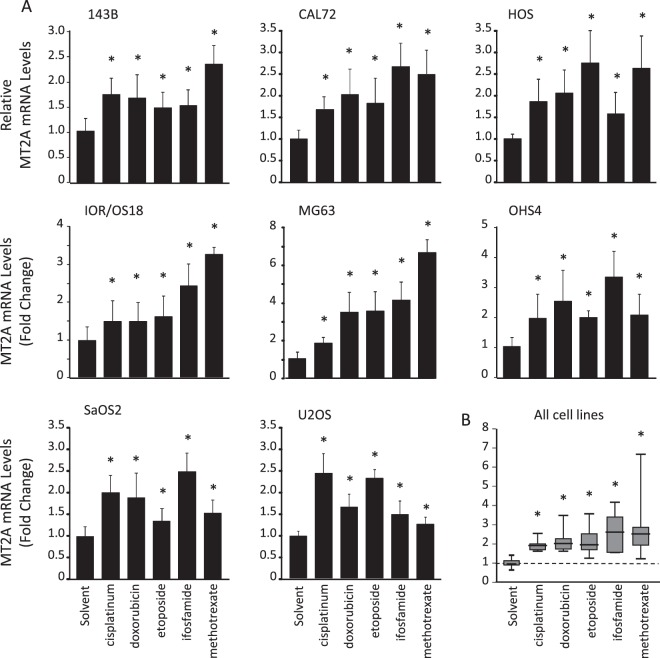
Chemotherapy induces MT2A neo-synthesis. (**A**) Expression pattern of MT2A in 143B, CAL72, HOS, IOR/OS18, MG63, OHS4, SaOS2, and U2OS cells incubated for 24 hrs in the presence of the chemotherapeutic drugs (100 µM cisplatinum; 1.84 µM doxorubicin; 10 µM etoposide; 5 mM ifosfamide or 5 mM methotrexate) or solvent, as assessed by RT-qPCR. GAPDH was used as internal reference gene. The relative mRNA level was calculated using the 2^−ΔΔCT^ method and expressed as mean ± standard deviation (n = 4). An asterisk (*) indicates a statistically significant difference (*p* < 0.05 *vs*. control). (**B**) Box plot of distribution of MT2A mRNA level in all cell lines incubated for 24 hrs in the presence of indicated drugs.

To evaluate the up-regulation of MT2A level by chemotherapy, cells were exposed to cisplatinum, doxorubicin, etoposide, mafosfamide, methotrexate or solvent for 24 hrs, and MT2A mRNA level was determined by RT-qPCR. All tested drugs induced an about 2-fold up-regulation of MT2A mRNA level in all tested cell lines (Fig. 2).

Then, to evaluate the possible involvement of glutathione (GSH), which is another key contributor to xenobiotic detoxication, cells were exposed to chemotherapeutic drugs for 24 hrs, and GSH level was determined using a colorimetric assay. Only etoposide induced a decrease in intracellular GSH level in all cell lines (−26 to −70%, p < 0.01 *vs*. solvent; Supplemental Fig. 1), whereas all other tested drugs did not affect GSH content.

Taken together, these results indicate that osteosarcoma cells rather promote MT2A neo-synthesis than trigger the glutathione-dependent detoxication in response to cisplatinum, doxorubicin, mafosfamide, or methotrexate. Etoposide exposure led to both MT2A induction and GSH efflux.

### MT2A level does not correlate with osteosarcoma cell migration ability

We determined the migration speed of our panel of osteosarcoma cell lines both in basal culture conditions (Supplemental Fig. 2A), and combined them to MT2A mRNA level (Supplemental Fig. 2B). No correlation was detectable (R < 0.2; p > 0.5).

These results indicate that MT2A level did not influence cell migration rate.

### MT2A silencing does not impact osteosarcoma cell behaviour

We established new stable cell lines modified by lentiviral transduction of shRNA sequences (Supplemental Fig. 3A), and investigated the impact of a repression of MT2A on cell behaviour. Cell morphology, spreading and cellular body size (Supplemental Fig. 3B–E), as well as cell proliferation rate as assessed by BrdU incorporation assay (Supplemental Fig. 3F) were comparable among the modified and parental cell lines. As detected in parental U2OS cells, the intracellular GSH level was unaffected by chemotherapy, except by etoposide (Supplemental Fig. 3G), in a similar manner in shControl and shMT2A U2OS cells.

These results indicate that silencing MT2A expression does not affect cell behaviour in basal culture conditions.

### MT2A silencing reinforces the cytotoxic effects of chemotherapy drugs

To investigate the impact of a repression of MT2A on cell response to chemotherapy, we established new stable cell lines modified by lentiviral transduction of shRNA sequences. We investigated four cell lines exhibiting the highest IC50 to chemotherapy among the tested panel of osteosarcoma cell lines, namely IOR/OS18, CAL72, U2OS and OHS4 (Supplemental Table 2). As expected, the cells modified with sh-MT2A sequences exhibited significantly lower MT2A mRNA level (−50 to −70% *vs*. shControl; Supplemental Fig. 4). We then evaluated the impact of lowered MT2A level on the dose-response to cisplatinum, doxorubicin, etoposide or ifosfamide. Cells in exponential phase of growth were exposed for 72 hrs to the indicated drugs. The MT2A-silenced cells exhibited a higher sensitivity to a given drug, as reflected by lower half maximal inhibitory concentration (IC50) values as compared to shControl cells (Table 1).

**Table 1:** Half maximal inhibitory concentrations (IC50) for chemotherapy.

	CAL72		IOR/OS18		OHS4	
	Control cells	shMT2A cells	Control cells	shMT2A cells	Control cells	shMT2A cells
cisplatinum	4.27 µM	2.95 µM	31.5 µM	5.27 µM	10.8 µM	3.37 µM
doxorubicin	0.07 µM	0.03 µM	0.16 µM	0.07 µM	0.12 µM	0.09 µM
etoposide	0.48 µM	0.32 µM	4.21 µM	2.35 µM	2.75 µM	1.43 µM
ifosfamide	27.4 µM	8.20 µM	30.3 µM	4.51 µM	2.43 µM	0.79 µM

Cell viability was assessed in human CAL72, IOR/OS18 and OHS4 cell lines incubated for 72 hrs in the presence of increasing doses of each indicated drug.

To evaluate if repression of MT2A can reverse chemo-resistance, we modified HOS cells that have acquired *in vitro* resistance to doxorubicin (Resistance Index = 88; see Methods section)^9^. This HOS-Doxo^R^ cell line exhibited a cross-resistance to etoposide (RI = 140) but not to cisplatin or ifosfamide^9^. As expected, Hos-Doxo^R^ cells modified with sh-MT2A sequences exhibited significantly lower MT2A mRNA level (−40% *vs*. shControl; Supplemental Fig. 4C). The MT2A-silenced cells exhibited a higher sensitivity to a given drug, as reflected by lower IC50 values as compared to shControl cells (Table 2).

**Table 2:** Half maximal inhibitory concentrations (IC50) for chemotherapy.

	HOS	HOS Doxo^R^	
	Parental cells	Control cells	shMT2A cells
cisplatinum	3.17 µM	2.42 µM	0.49 µM
doxorubicin	0.05 µM	4.39 µM	0.02 µM
etoposide	0.64 µM	90.5 µM	0.39 µM
ifosfamide	9.81 µM	10.5 µM	1.89 µM

Cell viability was assessed in human HOS Doxo^R^ incubated for 72 hrs in the presence of increasing doses of each indicated drug.

Taken together, these results indicate that silencing MT2A expression reinforces the cell sensitivity to chemotherapeutic drugs, and can reverse cell chemo-resistance.

### MT2A silencing promotes anti-tumour effects of chemotherapy in Xenograft models

**Figure 3:**
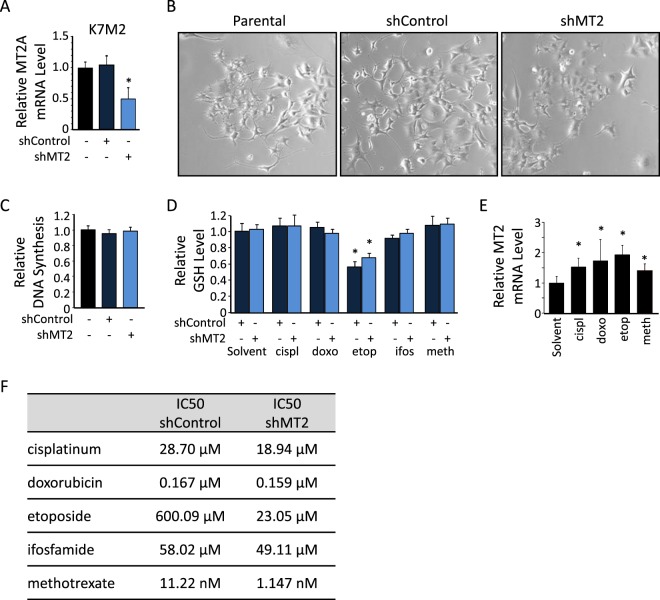
Characterization of MT2-silenced murine cell line. (**A**) Expression levels of MT2 in parental and stably modified K7M2 cells, as assessed by RT-qPCR. GAPDH was used as internal reference gene. The relative mRNA level was calculated using the 2^−ΔΔCT^ method and expressed as mean ± standard deviation (n = 3). An asterisk (*) indicates a statistically significant difference *vs*. shControl group (*p* < 0.05). (**B**) Representative images of parental, and shControl- or shMT2-modified K7M2 cells (200X magnification). (**C**) Relative DNA synthesis, as assessed by a BrdU incorporation colorimetric assay. Results are expressed as fold change value normalized to parental cells (mean ± standard deviation; n = 8). (**D**) Relative glutathione (GSH) level in shControl- or shMT2-modified K7M2 cells incubated in the presence of the indicated drugs, as assessed by a colorimetric assay. Results are expressed as fold change value normalized to untreated cells (mean ± standard deviation; n = 4). (**E**) Expression level of MT2 in parental K7M2 cells incubated for 24 h in the presence of the indicated drugs, as assessed by RT-qPCR. An asterisk (*) indicates a statistically significant difference *vs*. solvent (*p* < 0.05). (**F**) Half maximal inhibitory concentrations (IC50) for chemotherapy. Cell viability was assessed in murine K7M2 cell line incubated for 24 hrs in the presence of increasing doses of each indicated drug.

We performed similar experiments with the murine osteosarcoma cell line K7M2. Stable transduction of shRNA sequences targeting MT2 gene resulted in significant decrease in MT2 mRNA level, as compared to shControl or parental cells (−55%, p < 0.05; Fig. 3A). No significant modification in cell morphology or cell proliferation rate was detectable among the modified and parental cell lines (Fig. 3B,C, respectively). As observed in human cell lines, (i) only etoposide reduced GSH content in both shControl and shMT2 modified K7M2 cells (Fig. 3D), (ii) all tested chemotherapy drugs induced MT2 mRNA expression in parental K7M2 cells (Fig. 3E), and (iii) MT2-silenced cells exhibited a higher sensitivity to a given drug, as reflected by lower IC50 values as compared to shControl cells (Fig. 3F).

Taken together, these results indicate that the murine K7M2 cell line behaves the same as human cell lines and represents a suitable model for further *in vivo* investigations.

**Figure 4:**
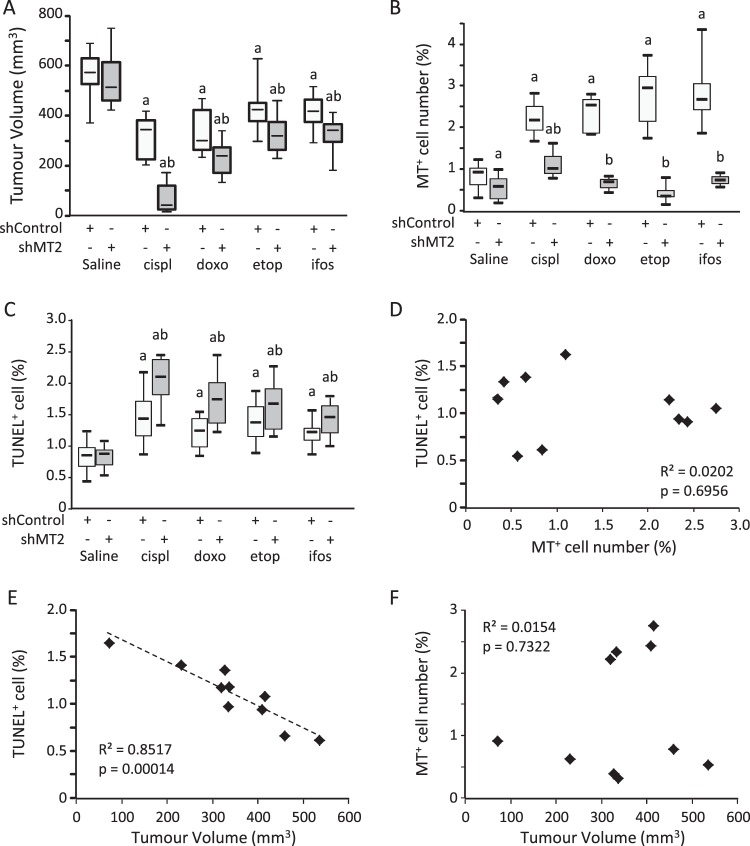
Silencing of MT2 improves chemotherapy cytotoxic effects in a preclinical model. K7M2 cells modified with shControl or shMT2 were injected intramuscularly into the thighs of SCID mice and administered with chemotherapy as described in the Methods section. (**A**) Boxplot specifying the tumour volume at dissection. a indicates a statistically significant difference *vs*. saline (*p* < 0.05); b indicates a statistically significant difference *vs*. shControl group (p < 0.05). (**B**) Boxplot specifying the MTs staining intensity in non-necrotic area. (**C**) Boxplot specifying the apoptotic cells as demonstrated by TUNEL staining in non-necrotic area. (**D**) Spearman correlation between the number of TUNEL positive cells, and number of MTs positive cells. The determination coefficient R² and the significance of the F test (P-value) are also given. (**E**) Spearman correlation between the number of TUNEL positive cells, and tumour volume. The black line shows the regression line. The determination coefficient R² and the significance of the F test (P-value) are also given. (**F**) Spearman correlation between the number of MTs positive cells, and tumour volume. The determination coefficient R² and the significance of the F test (P-value) are also given.

We injected shControl and shMT2 K7M2 cells in SCID mice, and then when tumours emerged we administrated chemotherapy regimen (cisplatinum, doxorubicin, etoposide or ifosfamide) or saline solution (vehicle) as described in the Methods section. As expected, the average tumour volume in mice injected with shControl cells was reduced by chemotherapy: from −25% with etoposide (p = 0.00046 *vs*. saline; Fig. 4A); −29% with ifosfamide (p < 0.0001); −42% with doxorubicin (p < 0.0001) to −45% with cisplatinum (p < 0.0001). No difference was detected in the average tumour volume between shMT2 and shControl groups without treatment (saline; p > 0.1). The tumour volume was more drastically reduced by chemotherapy regimens in the shMT2 group, as compared to treated mice bearing shControl cells-derived tumour: from −40% with etoposide (p = 0.0028 *vs*. shControl); −41% with ifosfamide (p = 0.0058); −58% with doxorubicin (p = 0.0033) to −87% with cisplatinum (p < 0.0001).

**Figure 5:**
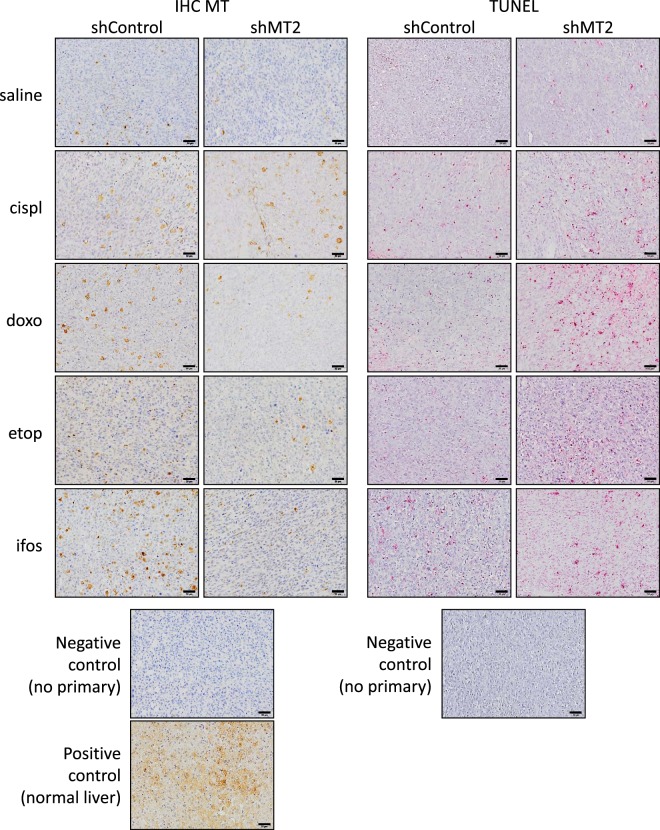
Silencing of MT2 improves chemotherapy cytotoxic effects in a preclinical model. Representative image showing immune-histochemical (IHC) staining for MTs in primary tumour FFPE sections (left panel). A negative control of the MTs stain by omitting the primary antibody, and a positive control of MTs stain on normal liver sample were included. The scale bars represent 50 μm (right panel). Representative images illustrating TUNEL staining in primary tumour FFPE sections. A negative control of TUNEL stain by omitting the primary antibody was included.

MT expression was assessed by IHC staining of FFPE (formalin-fixed paraffin-embedded) tumour sections (Fig. 5). As expected, the MT protein level in mice injected with shControl cells was induced by chemotherapy: from 2.7-fold with cisplatinum (p = 0.0384 *vs*. saline; Fig. 4B); 2.9-fold with doxorubicin (p = 0.0005); 3-fold with ifosfamide (p = 0.0001); to 3.4-fold with etoposide (p < 0.0001). As expected, the average signal was reduced in untreated (saline) shMT2 group as compared to shControl group (−32%, p = 0.0074), and markedly reduced in all treated conditions: from −57% with cisplatinum (p = 0.0248); −72% with doxorubicin (p = 0.0127); −85% with etoposide (p < 0.0001) to −86% with ifosfamide (p < 0.0001).

Cell death was assessed by TUNEL (terminal deoxynucleotidyl transferase dUTP nick end labelling) staining of FFPE tumour sections (Fig. 5). As expected, the percentage of TUNEL positive cells in shControl cells-derived tumour was increased by chemotherapy: from +41% with ifosfamide (p = 0.0012 *vs*. saline; Fig. 4C); +45% with doxorubicin (p = 0.00018); +68% with etoposide (p = 0.0003) to +76% with cisplatinum (p = 0.0034). No difference was detected in the percentage of TUNEL positive cells between shMT2 and shControl groups without treatment (saline; p > 0.1). The average TUNEL positive cells percentage was more drastically increased by chemotherapy regimens in the shMT2 group, as compared to treated mice bearing shControl cells-derived tumours: from +77% with ifosfamide (p = 0.0188 *vs*. shControl); 2-fold with etoposide (p = 0.0028); 2.2-fold with doxorubicin (p = 0.042) to 2.5-fold with cisplatinum (p = 0.0043).

Overall, the TUNEL positive cell number negatively correlated with MT positive cell number and tumour volume (R² > 0.5; p < 0.05; Fig. 4D,E). In contrast, no correlation was detected between MT2 level and tumour volume (R² < 0.1, p > 0.7; Fig. 4F).

**Figure 6:**
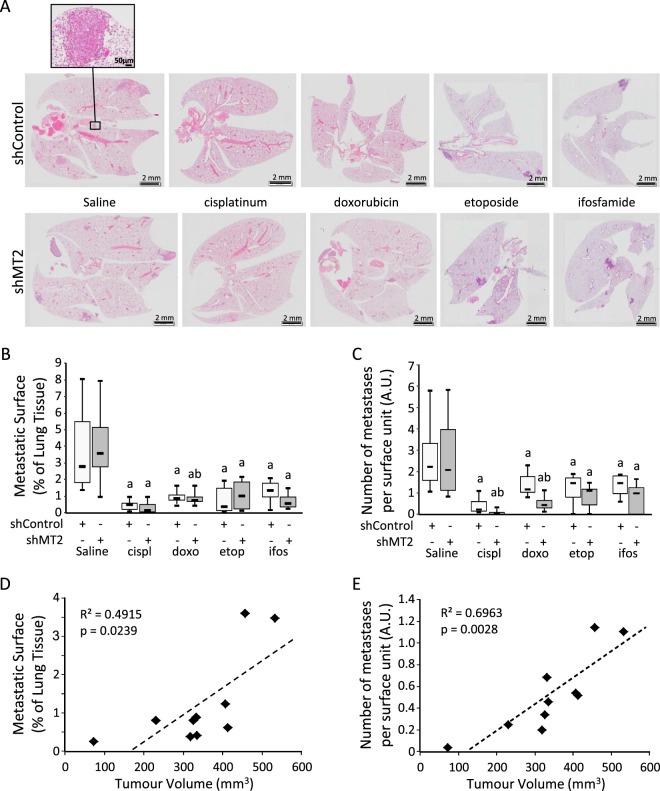
Silencing of MT2 influences chemotherapy tumour pulmonary metastatic dissemination. K7M2 cells modified with shControl or shMT2 were injected intramuscularly into the thighs of SCID mice and administered with chemotherapy as described in the Methods section. (**A**) H&E staining of lung tissue sections. The scale bars represent 2 mm, except in the upper-left insert: Scale bar  = 50 µm. (**B**) Boxplot specifying the metastatic surface. Results are expressed as percent of whole pulmonary tissue. a indicates a statistically significant difference *vs*. saline (*p* < 0.05); b indicates a statistically significant difference *vs*. shControl group (p < 0.05). (**C**) Boxplot specifying the number of metastatic nodules per field. a indicates a statistically significant difference *vs*. saline (*p* < 0.05); b indicates a statistically significant difference *vs*. shControl group (p < 0.05). (**D**) Spearman correlations between the relative metastatic surface, as expressed as percent of whole pulmonary tissue, and tumour volume as expressed as mm^3^. The black line shows the regression line. The determination coefficient R² and the significance of the F test (P-value) are also given. (**E**) Spearman correlations between the number of metastatic nodules per field, and tumour volume.

Pulmonary metastatic foci were assessed by HES staining of FFPE lung sections (Fig. 6A). As expected, the average metastatic surface was markedly reduced by chemotherapy regimens in the mice injected with shControl cells: from −60% with ifosfamide (p = 0.037 *vs*. saline; Fig. 6B); −61% with doxorubicin (p = 0.036); −76% with etoposide (p = 0.0042) to −87% with cisplatinum (p = 0.0031). No difference could be detected in the average metastatic surface at sacrifice between shMT2 and shControl groups without treatment (saline; p > 0.3). The average metastatic surface was not more drastically reduced in the mice injected with shMT2 cells, as compared to mice injected with shControl cells when treated with chemotherapy (p > 0.2).

The average number of metastases was markedly reduced by chemotherapy regimens in the mice injected with shControl cells (−38% to −79%, p < 0.01; Fig. 6C), and more drastically reduced in the mice injected with shMT2 cells by cisplatinum (−96%, p = 0.00045 *vs*. saline) or doxorubicin (−77%, p = 0.0013), whereas no difference could be detected between shMT2 and shControl groups without treatment (saline; p = 0.335).

Overall, the metastatic surface and the number of metastases positively correlated with tumour volume (Fig. 6D,E, respectively).

Taken together, these results confirm that MT2A level influence chemotherapy induced-cell death, and primary tumour development resulting in modulation of pulmonary metastatic dissemination.

## Discussion

Chemo-resistance is a major obstacle to therapeutic success of osteosarcoma. Although neo-adjuvant chemotherapy has considerably improved the prognosis of osteosarcoma patients since the past four decades, about two out of five patients will experience relapse or/and recurrence within five years after diagnosis.

In the present study, we showed a strong positive correlation between MT2A/MT2 mRNA level and cell sensitivity to a panel of chemotherapeutic drugs currently used in clinical practice^10^, as assessed by half maximal inhibitory concentration (IC50) values. These data are consistent with the correlation we previously reported between MT2A mRNA level in chemo-naive biopsy, and tumour response to chemotherapy thereafter (based on Huvos scoring of tumour necrosis), and overall survival of patients^7^. Taken together, our data showed that MT2A contributes to chemotherapy resistance in osteosarcoma, and could be considered as a predictive indicator at diagnosis for osteosarcoma responsiveness to current chemotherapy regimen, whatever their mechanism of action.

We also investigated the possible role of MT2A in metastatic dissemination. We did not detect any correlation between MT2/MT2A level and *in vitro* cell motility using a panel of cell lines. These data are consistent with our previous study reporting no modification in cell motility or invasion capacity of MT2A-overexpressing cells or MT2A-silenced cells compared to parental cells^7^. Moreover, we did not detect any influence of MT2 silencing on primary tumour spreading to the lungs in our Cell line-Derived Xenograft (CDX) model, either in the average metastatic foci surface or number, suggesting that MT2A level may only influence cell responsiveness to toxics and not the invasion process.

In response to chemotherapeutic drugs, cancer cells could adopt several strategies to develop their resistance such as the control of the transmembrane entry/efflux of the compounds; the activation of DNA repair machinery (ERCC1 and 2, NER genes…); the adaptation of the drug target (dihydrofolate reductase (DHFR), topoisomerase…); or the synthesis of detoxication proteins (gluthation-S-transferase (GST), metallothioneins…) for example (reviewed in^11^). Such adaptive behaviour has been reported in osteosarcoma. Indeed, an up-regulation of DHFR and MDR1 (multidrug resistance protein 1) have been reported to be associated with an enhanced chemo-resistance to methotrexate and doxorubicin^12^. Increased expression of P-glycoprotein in tumour cells was significantly associated with poor survival in patients with osteosarcoma^13–16^. An overexpression of GSTπ, Hsp27, LRP (lung resistance-related protein) as well as MTs has been detected in osteosarcoma tumours with poor histologic response to pre-operative chemotherapy^17^. However, no statistical differences could be detected in terms of response to treatment and patient outcome. This apparent contradiction with our data could be related to the specificity of the molecular tools. Almost all studies focused on an immuno-histochemical detection of MTs in FFPE tissue samples. However, the MTs antibodies that are commonly used for such IHC staining do not distinguish between the different highly homologous isoforms of the MT superfamily. This could minimize the impact of the variations of one or a few of the isoforms.

In our panel of osteosarcoma cell models, IC50 values were heterogeneous from one cell line to another, but overall correlated to the MT2/MT2A mRNA level. We also observed that all tested drugs induce MT2/MT2A expression, independently of the genomic alterations detectable in the cell lines. Such adaptive process could explain why tumours become resistant as cycles of chemotherapy progress. The evaluation of MT2A mRNA level would inform about the innate resistance status when measured at diagnosis, and about the acquired resistance linked to an adaptive process if monitored during treatment.

We also focused here on the possible complementary role of the glutathione pathway in the observed MT2A-related chemo-resistance. GST catalyses the conjugation of toxic electrophilic chemicals to the tri-peptide glutathione (GSH) in order to facilitate their rapid clearance from the cell^18^. Increased GST expression has been associated with a resistant phenotype to conventional chemotherapy in different cancer types (reviewed in^19^). Some GST polymorphisms are correlated to clinical outcome of osteosarcoma patients^20^. In our panel of osteosarcoma cell lines we did not observe any variation in intracellular GSH levels after exposure to chemotherapy with the exception of a decrease for etoposide, that is known to undergo glutathione conjugation^21^ and to increase GSH export^22^. In addition, the GSH levels are comparable in cells modified with non-relevant shRNA sequences or silenced for MT2/MT2A, even under chemotherapy treatment. Taken together, these data are consistent with our hypothesis that the detoxication process triggered by osteosarcoma cells incubated in the presence of chemotherapeutic drugs is mainly metallothionein-dependent.

The second significant finding of our study is the proof of concept that MT2A could also be a valuable therapeutic target to improve osteosarcoma sensitivity to chemotherapy. We showed that MT2/MT2A silencing enhanced cytotoxic action of current chemotherapeutic drugs on osteosarcoma cells *in vitro*, reflected by the lowering of IC50 values for all tested drugs. More importantly, we showed that MT2 silencing led to higher cytotoxic effects of drugs in preclinical Cell line-Derived Xenograft (CDX) models. This fully confirmed our hypothesis that MT2 targeting rendered tumours significantly more sensitive to chemotherapy. Taken together, these data show that MT2A could be considered as a therapeutic target to prevent or reduce osteosarcoma resistance to chemotherapy. Not many strategies of targeting MT isoforms have reached the clinical practice as therapeutic agents (reviewed in^6^). Further investigations are required to identify a physiological and clinically safe strategy to prevent MT2A neo-synthesis under treatment or to reduce MT2A levels in case of high basal content.

In summary, this study identified the key role of high MT2A expression level in the resistance of osteosarcoma cells to chemotherapeutic drugs. In effect, we revealed a negative correlation between MT2A level and drug efficacy (IC50 values), and anti-tumour effects for various chemotherapeutic drugs when cells were silenced for MT2A. Our results thus support a model whereby MT2A contributed to lowering the drug cytotoxic effects and could be a valuable therapeutic target to improve osteosarcoma sensitivity to chemotherapy. In conclusion, we demonstrated that MT2A could be considered as a predictive biomarker of the efficacy of chemotherapy for patients with osteosarcoma, and as a potential therapeutic target to develop novel treatment strategy to prevent or de-escalate chemo-resistance in osteosarcoma.

## Methods

### Cell lines and cell culture

The human osteosarcoma cell lines 143B, HOS, MG63, SaOS-2, U2OS, and the murine cell line K7M2 were originally obtained from ATCC (LGC Standards Sarl; Molsheim, France). The human cell line IOR/OS18 was kindly provided by Prof M Serra (Istituti Ortopedici Rizzoli; Bologna; Italy). The human cell line CAL72 was kindly provided by Dr N Rochet (Institute of Biology Valrose, Nice, France)^23^. The human cell line OHS-4 was kindly provided by Drs Fournier and Price^24^. The human HOS-Doxo^R^ cell lines was established by continual exposure to increasing concentration of Doxorubicin (from 0.01 µM up to 1.3 µM)^9^.

All the cells were cultured in Dulbecco’s Modified Eagle’s Medium (DMEM) supplemented with 10% heat inactivated Fœtal Calf Serum, at 37 °C in a 5% CO2 humidified atmosphere. The culture media were replaced three times a week.

### Lentiviral transduction

Cells were seeded at a density of 25,000 cells/cm². The next day, cells were transduced with lentiviral particles encoding human sh-MT2A, murine sh-MT2 or non-targeting (control) shRNA sequences purchased from Santa Cruz Biotechnology (Santa Cruz, CA, USA), according to the manufacturer’s instructions. Virus-containing medium was changed with fresh medium 24 hrs after infection. Four days later, stably modified cells were selected with 10 µg/ml puromycin dihydrochloride (Sigma-Aldrich) for 3 days. Viable cells were then routinely maintained in complete medium.

### *In vitro* cell viability assay

Cells were seeded at a density of 15,000 cells/cm², and the next day treated with the indicated drug for 24 or 72 h. Cell viability was evaluated by the MTS tetrazolium assay (CellTiter 96 aqueous one solution cell proliferation assay kit; Promega, Charbonnieres, France), according to the manufacturer’s instructions.

The half-maximal inhibitory concentration IC50 was interpolated from sigmoidal dose-responses curves (IC50 Calculator software; AAT Bioquest, Sunnyvale, CA, USA).

### Wound healing assay

The cell migration rate was assessed using the IncuCyte Live Cell Imaging system according to the manufacturer’s instructions (Essen Bioscience Ltd., Birmingham, UK). Cells monolayers were scratched, then washed once and maintained in culture with phase-contrast photographs collected every 4 hrs for 72 hrs.

### Glutathione assay

The level of total glutathione (GSSG + GSH) was assessed using glutathione assay kit (Sigma-Aldrich), according to the manufacturer’s recommendations.

### RNA extraction

The RNA was extracted using TRIzol Reagent (Thermo Fisher Scientific, Illkirch, France) according to the manufacturer’s protocol, and stored at −80 °C in RNase-free water. Purity and quantity of RNA samples were assessed using the NanoVue Plus spectrophotometer (GE Healthcare Life Sciences, Velizy-Villacoublay, France).

### Quantitative real-time RT-PCR

Total RNA (1 µg) were denatured for 10 min at 70 °C then incubated at 37 °C for 90 min in a buffer containing MMLV reverse transcriptase (10U), DTT (dithiothreitol; 3 mM), oligodT primers (0.05 µM) and dNTPs (deoxynucleotide mix; 1 mM). A final step of inactivation of the enzyme is carried out at 95 °C for 5 min.

Quantitative PCR was performed on ViiA7 real-time PCR system (Thermo Fisher Scientific) using SYBR Green Master kit (Thermo Fisher Scientific) supplemented with 0.5 µM of specific primers (Supplemental Table 1). Thermal conditions were: activation for 15 min at 95 °C then 40 cycles of denaturation at 95 °C for 20 sec, 59 °C annealing for 15 sec and 72 °C extension for 15 sec. Upon completion of each run, a melting curve analysis was performed to check specificity of the primers. All signals with threshold cycle (Ct) > 36 were set as undetermined. The relative gene expression was calculated using the 2^−∆∆Ct^ method.

### Xenograft models

All procedures were conducted according to the guidelines formulated by the European Commission for experimental animal use (L358-86/609EEC), and with the approval of the French local ethical animal committee (Comité d’éthique en Expérimentation Animale n°26/CEEA26, Villejuif, France). Severe combined immunodeficiency (SCID) female mice (Charles River, Arbresle, France) of 5-weeks old were randomized, housed at 22 °C to 24 °C with a 12-hour light/dark cycle and maintained in a pathogen-free enriched environment throughout the experiment. They were fed with a standard mice pellet diet and had free access to water. After acclimatization for 7 days, mice were injected intramuscularly (IM) with K7M2 cells (10^6^ cells/15 µl PBS) in both thighs under isoflurane/air inhalational anaesthesia. After 10 days allowing tumour growth, mice were treated by intraperitoneal injection of cisplatinum (7 mg/kg body weight), doxorubicin (4 mg/kg body weight), etoposide (5 mg/kg body weight) or ifosfamide (100 mg/kg body weight) every 5 days. A vehicle-treated (saline) group was included. At day 28, mice were killed by CO_2_ asphyxiation. Tumour diameters were measured with digital callipers, and the tumour volume in mm^3^ was calculated by the formula = width × height × length × (π/6). Tumours and lungs were fixed in 4% paraformaldehyde in PBS before paraffin embedding.

### Immunohistochemistry

Formalin-fixed paraffin embedded (FFPE) tumours or lungs sections (4 µm thick) were deparaffinised in xylene and rehydrated through a graded series of ethanol before haematoxylin & eosin (H&E) staining.

*In situ* cell death detection was performed on tumour sections by TUNEL (terminal deoxynucleotidyl transferase-mediated dUTP nick-end labelling) assay according to the manufacturer’s recommendation (Roche Diagnostics, Boulogne-Billancourt, France). Slides were revealed with permanent Red (Dako-Agilent Technologies, Courtabœuf, France) and counterstained with haematoxylin.

Immuno-histological detection of MTs and Ki67 was performed on tumour sections. After immersion in 3% H_2_O_2_ for 10 min to block endogenous peroxidase activity, and an antigen retrieval heating step, with a citrate buffer (pH 6), at 98 °C for 30 min, slides were incubated for 10 min with blocking reagent (Klear Mouse HRP; GBI Labs, Bothell, WA, USA) to block nonspecific staining, and incubated for 60 min with mouse monoclonal anti-mouse metallothionein antibody (clone E9, Dako-Agilent Technologies) at a dilution of 1:200, or with anti-Ki67, 1:200 (Neomarkers, LabVision, Microm Microtech, Francheville, France). After two rinses in wash buffer, slides were incubated successively in an enhancer antibody and the polymer HRP anti-mouse or anti-rabbit, respectively for 30 min. Slides were washed and incubated for 5 min with DAB (diaminobenzidine) as peroxidase substrate (Thermo Fisher Scientific). After rinsed by tap water, the slides were counterstained with haematoxylin.

Each slide was digitalized using an Olympus VS120 slide scanner. Quantitation of MT, and TUNEL positive cells was performed according to^25^, based on image analysis software (ImageJ 1.52 h, National Institutes of Health, Bethesda, Maryland, USA).

### Statistical procedures

The data were analysed by two-factor analysis of variance (ANOVA) with the statistical package super-ANOVA. The correlation coefficients were obtained by Spearman correlation analysis. Preclinical data was analysed with the non-parametric Mann-Whitney test. Paired samples were analysed with the non-parametric Wilcoxon signed-rank test. *P* value of less than 0.05 was considered statistically significant.

## Supplementary information

supplementary informations

## Data Availability

Data sharing is not applicable to this article as no datasets were generated or analysed during the current study.
