# Supplementary material for: MT2A is an early predictive biomarker of response to chemotherapy and a potential therapeutic target in osteosarcoma
Source: Sci Rep. 2019 Aug 23;9:12301. doi: 10.1038/s41598-019-48846-2 (PMC6707240; doi:10.1038/s41598-019-48846-2)
Supplement: Supplementary file 1 — supplementary informations [file 41598_2019_48846_MOESM1_ESM.pdf]

Supplementary materials

MT2A is an early predictive biomarker of response to chemotherapy and a potential therapeutic target in osteosarcoma

Adèle Mangelinck[1,2,3], Maria Eugénia Marques da Costa[4,5,6], Bojana Stefanovska[1,5], Olivia Bawa[7], Mélanie Polrot[7], Nathalie Gaspar[4,8], and Olivia Fromigué[1,5,*]

[1] INSERM, UMR981, Gustave Roussy, Villejuif, F-94805, France

[2] Ecole Nationale Supérieure de Chimie de Montpellier (ENSCM), Montpellier, F-34090, France

[3] Université de Montpellier, Montpellier, F-34090, France

[4] CNRS, UMR8203, Gustave Roussy, Villejuif, F-94805, France

[5] Université Paris Sud, Université Paris Saclay, Orsay, F-91400, France

[6] CESAM, Department of Biology, University of Aveiro, Aveiro, P-3810, Portugal

[7] Plateforme d'évaluation préclinique (PFEP), Gustave Roussy, Villejuif, F-94805, France

[8] Département de cancérologie de l'enfant et de l'adolescent, Gustave Roussy, Villejuif, F-94805, France

* Corresponding author: olivia.fromigue@inserm.fr

Supplemental Table 1: Primer sequences used for qPCR on human (h) and murine (m) samples.

**Name**	**GeneBank No**	**Primer sequence (5' – 3')**
hGAPDH	NM_002046	Forward AGCCACATCGCTCAGACAC<br>Reverse GCCCAATACGACCAAATCC
hMT2A	NM_005953	Forward TGCACCTCCTGCAAGAAA<br>Reverse CAGCAGCTGCACTTGTCC
mGAPDH	NM_008084	Forward ACACATTGGGGGTAGGAACA<br>Reverse AACTTTGGCATTGTGGAAGG
mMT2	NM_008630	Forward CATGGACCCCAACTGCTC<br>Reverse AGCAGGAGCAGCAGCTTT

Supplemental Table 2. Half maximal inhibitory concentrations (IC50) for chemotherapy. Cell viability was assessed by the MTS test in the indicated human osteosarcoma cell lines incubated for 72 hrs in the presence of increasing doses of each indicated drug.

Cell Line	CISP (µM)	DOXO (µM)	ETOP (µM)	IFOS (µM)	MTX (µM)
143B	2.04	0.046	0.670	12.34	0.040
CAL72	14.27	0.201	2.836	27.42	0.298
HOS	3.17	0.049	0.638	9.814	0.019
IOR/OS18	31.54	0.159	4.21	30.32	0.327
MG63	2.23	0.140	3.41	11.12	0.327
OHS4	10.813	0.098	2.751	2.431	0.006
SaOS2	3.87	0.062	2.27	17.94	0.059
U2OS	9.40	0.155	2.28	23.05	0.029

SUPPLEMENTAL FIGURE 1

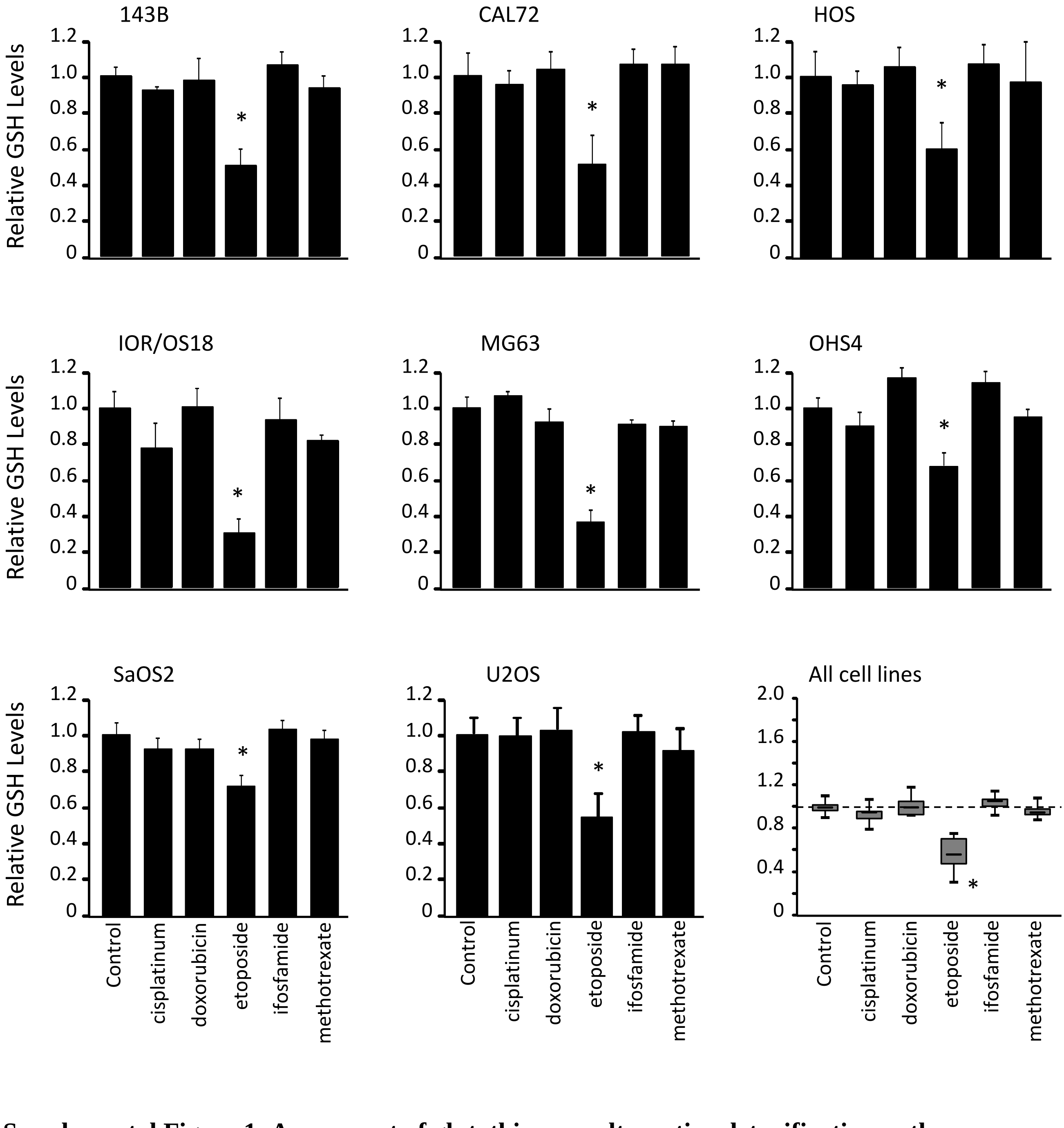

Supplemental Figure 1: Assessment of glutathione as alternative detoxification pathway. Relative glutathione (GSH) level in 143B, CAL72, HOS, IOR/OS18, MG63, OHS4, SaOS2, and U2OS cells incubated in the presence of the chemotherapeutic drugs (100 µM cisplatinum; 1.84 µM doxorubicin; 10 µM etoposide; 5 mM ifosfamide or 5 mM methotrexate) or solvent, as assessed by a colorimetric assay. Results are expressed as fold change value normalized to untreated cells (mean ± standard deviation; n = 6). An asterisk (*) indicates a statistically significant difference ($p < 0.05$). Box plot of distribution of relative GSH level in all cell lines incubated for 24 hrs in the presence of indicated drugs.

SUPPLEMENTAL FIGURE 2

A

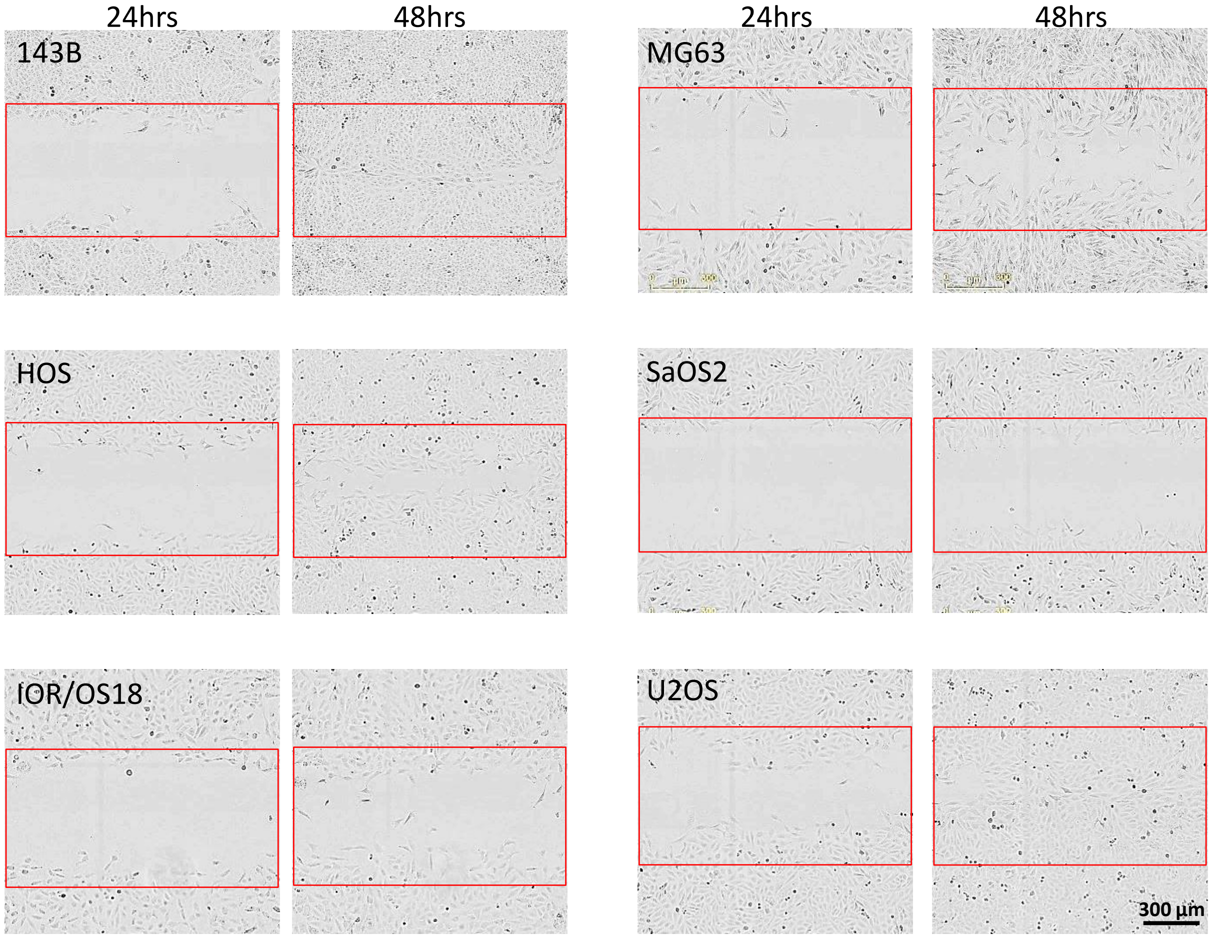

B

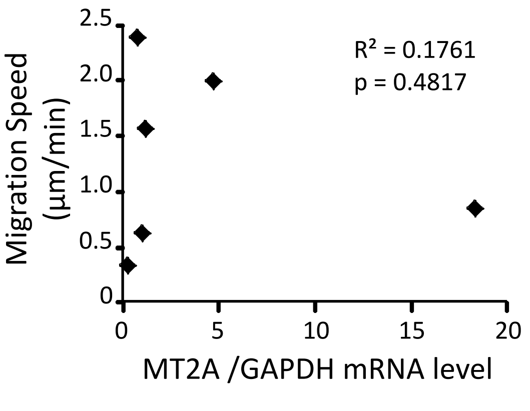

Supplemental Figure 2: MT2A level does not modulate cell migration. (A) Representative photographs of indicated cells migrating into scratch wound area at 24, and 48 hrs after scratch. Red lines indicate initial scratch wound area. (B) Spearman correlation between migration speed, and MT2A mRNA level in the indicated cell lines. The determination coefficient R^2 and the significance of the F test (P-value) are also given.

SUPPLEMENTAL FIGURE 3

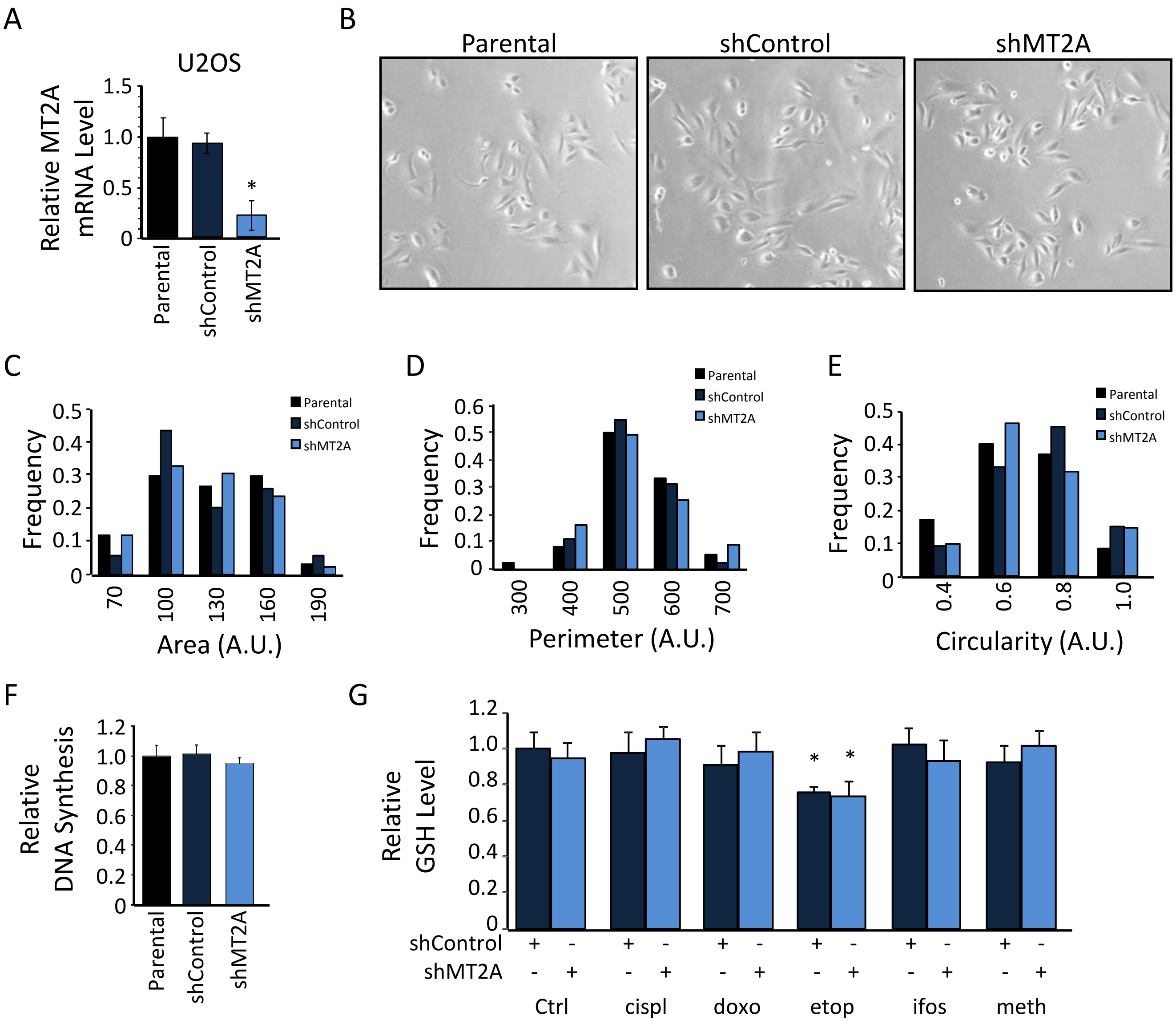

Supplemental Figure 3: Characterization of MT2A-silenced U2OS cell line. (A) Expression levels of MT2A in parental and stably modified U2OS cells, as assessed by RT-qPCR. GAPDH was used as internal reference gene. The relative mRNA level was calculated using the $2^{-\Delta\Delta CT}$ method and expressed as mean ± standard deviation (n = 3). An asterisk (*) indicates a statistically significant difference ($p < 0.05$ *vs*. control). **(B)** Representative images in parental and stably modified cells (200X magnification). **(C-E)** Distribution of cell shape parameters as function of the frequency of occurrence. **(F)** Relative DNA synthesis, as assessed by a BrdU incorporation assay. Results are expressed as fold change value normalized to parental cells (mean ± standard deviation; n = 8). **(G)** Relative glutathione (GSH) level in shControl- or shMT2A-modified U2OS cells incubated in the presence of the indicated drugs, as assessed by a colorimetric assay. Results are expressed as fold change value normalized to untreated cells (mean ± standard deviation; n = 4).

SUPPLEMENTAL FIGURE 4

A

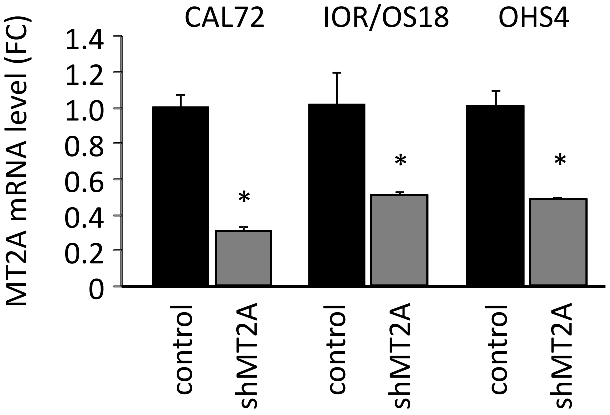

B

	CAL72		IOR/OS18		OHS4	
	Parental cells	shMT2A cells	Parental cells	shMT2A cells	Parental cells	shMT2A cells
cisplatinum	4.27 µM	2.95 µM	31.5 µM	5.27 µM	10.8 µM	3.37 µM
doxorubicin	0.07 µM	0.03 µM	0.16 µM	0.07 µM	0.12 µM	0.09 µM
etoposide	0.48 µM	0.32 µM	4.21 µM	2.35 µM	2.75 µM	1.43 µM
ifosfamide	27.4 µM	8.20 µM	30.3 µM	4.51 µM	2.43 µM	0.79 µM

C

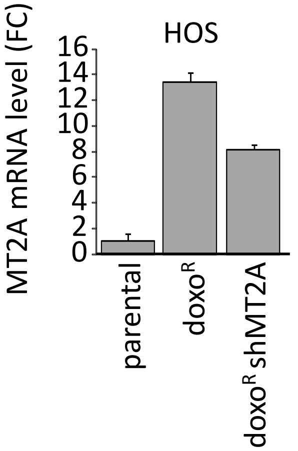

D

	HOS		
	Parental cells	Doxo-resistant cells	
		Control cells	shMT2A cells
cisplatinum	3.17 µM	2.42 µM	0.49 µM
doxorubicin	0.05 µM	4.39 µM	0.02 µM
etoposide	0.64 µM	90.5 µM	0.39 µM
ifosfamide	9.81 µM	10.5 µM	1.89 µM

Supplemental Figure 4: Characterization of MT2A-silenced human cell lines. (A) Expression levels of MT2A in parental and stably modified U2OS cells, as assessed by RT-qPCR. GAPDH was used as internal reference gene. The relative mRNA level was calculated using the $2^{-\Delta\Delta CT}$ method and expressed as mean ± standard deviation (n = 3). An asterisk (*) indicates a statistically significant difference (*p* < 0.05 *vs.* control).
(B,D) Half maximal inhibitory concentration (IC50) values determined after 72 hrs incubation in the presence of the indicated drugs.

SUPPLEMENTAL FIGURE 5

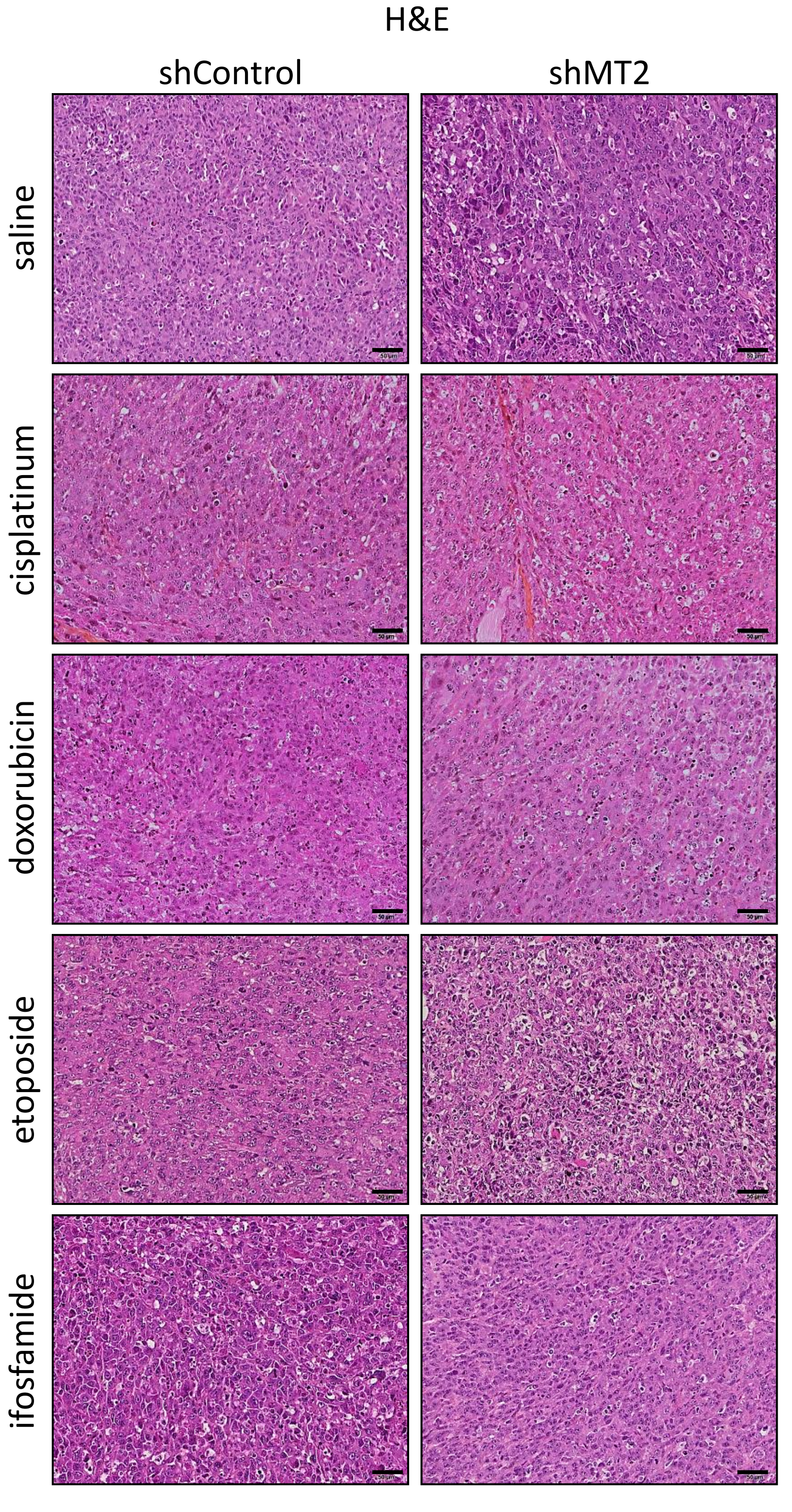

Supplemental Figure 5. K7M2 cells modified with shControl or shMT2 were injected IM into the thighs of SCID mice and administered with chemotherapy as described in the materials & methods section. Representative image showing Hematoxylin and Eosin (H&E) staining in K7M2 shControl- or shMT2-derived primary tumours. The scale bars represent 50 μm.

SUPPLEMENTAL FIGURE 6

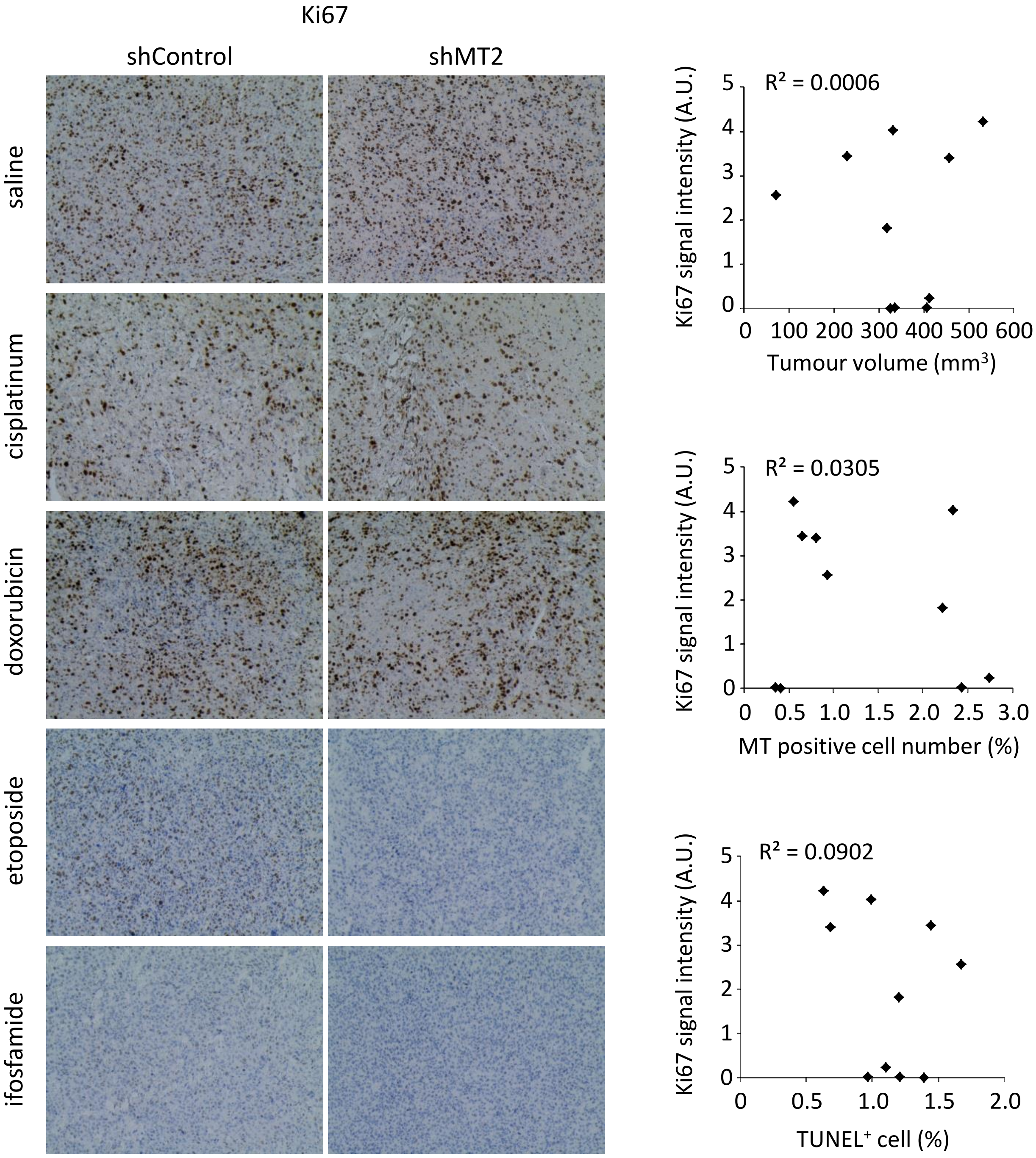

Supplemental Figure 6. K7M2 cells modified with shControl or shMT2 were injected IM into the thighs of SCID mice and administered with chemotherapy as described in the materials & methods section. Representative image showing immune-histochemical (IHC) staining for Ki67 in primary tumour FFPE sections.

Spearman correlations between the number of Ki67 positive cells, and tumour volume as expressed as mm3. The determination coefficient R^2 are also given.
